# Organ-specific bioaccumulation and deterministic health risk assessment of selected heavy metal(loid)s in commercial fish from a southern harbor region (Nagapattinam)

**DOI:** 10.3389/ftox.2026.1773891

**Published:** 2026-04-10

**Authors:** Suryapratap Ray, Rahul Vashishth

**Affiliations:** Department of Biosciences, School of Biosciences and Technology, Vellore Institute of Technology, Vellore, India

**Keywords:** bioaccumulation, contamination, food safety, heavy metals, ICP-MS

## Abstract

**Introduction:**

Heavy metal contamination of coastal waters results in bioaccumulation in fish, posing a significant route of human exposure via seafood consumption. The present study focuses on organ-specific metal distribution and associated health risk indices in three important fish species from the Nagapattinam coast, India.

**Methods:**

In the current study, three species of fish (*Nemipterus japonicus*, *Oreochromis mossambicus*, and *Lates calcarifer*) were considered with three biological replicates per species obtained from local fish markets of Nagapattinam, Tamil Nadu, India, during January–February 2024. The HM profiling was performed on three organs: liver, gills, and muscle tissues. HMs, including arsenic (As), cadmium (Cd), chromium (Cr), mercury (Hg), lead (Pb), strontium (Sr), and vanadium (V), were analyzed using inductively coupled plasma mass spectrometry (ICP-MS).

**Results:**

The concentrations were found to be in the range of 0.025–21.53 μg kg^−1^. Upon non-carcinogenic risk assessment, the target hazard quotient (THQ) for all species was found to be <1 for both adults and children, indicating low non-carcinogenic risk associated with the consumption of the selected species under the assumed intake scenario. In contrast, the cancer risk (CR) value for chromium in *Nemipterus japonicus* was found to be elevated (children: 1643.58 × 10^−6^ and adults: 939.19 × 10^−6^), assuming a conservative worst-case exposure scenario in which total chromium is considered as Cr(VI), potentially inflating cancer risk estimates.

**Discussion:**

Overall, the findings suggest low non-carcinogenic risk under average consumption conditions; however, the estimated carcinogenic risk for chromium exceeded the commonly referenced acceptable threshold under conservative assumptions and should therefore be interpreted with caution. These results highlight the need for further investigation, particularly chromium speciation analysis, to refine risk estimates for fish consumed from the Nagapattinam marketplace.

## 1 Introduction

Fish is a major dietary source of high-quality protein containing essential micronutrients and is highly relevant to global nutrition and food security. However, coastal ecosystems are sensitive to anthropogenic activities, making the marine organisms susceptible to heavy metal accumulation and subsequent human exposure through seafood consumption (Venkadeshwaran et al., 2025). Despite the importance of marine fisheries in India, data on heavy metal contamination and health risks in fish from Nagapattinam remain limited, highlighting the need for targeted risk assessment in this region (Perumal et al., 2023). In 2019–2020, India exported 1.289 million tonnes of marine products, valued at INR 466.63 billion. This includes a wide range of products, with frozen shrimp making up a significant share of the total export volume and value. The primary export markets include the USA, the European Union, and China (Kumar et al., 2019). Our study area, Nagapattinam in the state of Tamil Nadu (TN) (Figure 2), is one of the major harbors in TN. TN has 12 fishing harbors, nine major and three minor (Department of Fisheries, 2020). This infrastructure is vital for supporting the state’s fishing and export activities. Fish and fish products are essential to many parts of a healthy human diet, especially for people who are reluctant to consume red meat, have weakened immune systems, or are malnourished, pregnant, or breastfeeding. Fish is a good source of digestible protein and contains essential fat-soluble amino acids, vitamins, trace minerals, lipids, and omega-3 polyunsaturated fatty acids (Moiseenko and Gashkina, 2020; Morales, 2022; Sivaperumal et al., 2007). Furthermore, eating fish helps lower the risk of developing various diseases. The biochemical makeup of fish muscles can vary significantly depending on several factors, including species, age, sexual development, dietary region, sex, environment, season, and muscle type (Younis et al., 2021). The nutritional value of various fish species is not constant and varies seasonally (Avigliano et al., 2019). Fish is recognized as a high-quality protein source owing to the abundance, variety, digestibility, and significantly high biological value of its amino acid constituents. Fish organs are used in pollution biomonitoring; the liver is an important organ for the accumulation of heavy metals in fish metabolism (Ahmed et al., 2013). Muscle is not only essential for human nourishment but also an excellent way to assess the adverse effects of consuming heavy metals (Younis et al., 2021). In occurrences of heavy metal contamination, muscle tissue functions as a pivotal criterion for measuring health-associated hazards and is fundamental for human dietary demands. The gills get the largest amount of metal ions available in the surroundings and have a large surface area exposed to water at the same time. Non-essential metals such as cadmium (Cd), arsenic (As), and lead (Pb) are detrimental to biological systems and possess the potential to induce various diseases. Pb and Cd are heavy metals to which aquatic organisms exhibit hypersensitivity due to their significant capacity for bioaccumulation (Łuczyńska and Paszczyk, 2019). Harmful and undesirable metals like chromium [Cr(VI)] and lead (Pb) have no biological use and can even cause cancer (Bakhshalizadeh et al., 2022; Balali-Mood et al., 2021). Kidney failure is a consequence of chronic exposure to these metals, which render harmful effects on the kidneys, namely on several enzymes in the renal tubules involved in protein reabsorption (Makarenko et al., 2021).

Although several studies have reported heavy metal contamination in marine fish along different sectors of the Indian coastline (Copat et al., 2013; Avigliano et al., 2019; Noman et al., 2022; Zaghloul et al., 2024), most investigations have focused on limited species, single organs (primarily muscle), or a restricted number of metals. Furthermore, many studies focus primarily on concentration reporting without incorporating multi-metric human health risk assessment models, which are recommended for comprehensive exposure characterization (United States Environmental Protection Agency, 1989; European Food Safety Authority, 2009).

Importantly, Nagapattinam Harbor an ecologically sensitive and commercially active coastal zone influenced by intensive fishing activity, and localized anthropogenic pressure has not been systematically evaluated for simultaneous organ-specific bioaccumulation of multiple essential and non-essential metal(loid)s coupled with integrated risk modeling. Previous regional assessments in the Bay of Bengal have reported metal occurrence in selected matrices (Lakshmanasenthil et al., 2013; Silambarasan, 2016; Thiyagarajan et al., 2012), yet an integrated approach combining multi-organ bioaccumulation profiling with quantitative non-carcinogenic and carcinogenic risk characterization for local seafood consumers has not been comprehensively addressed. To date, no study has comparatively assessed liver, gill, and muscle accumulation patterns in commercially important fish species from this harbor ecosystem while applying internationally accepted non-carcinogenic and carcinogenic risk indices.

**FIGURE 1:**
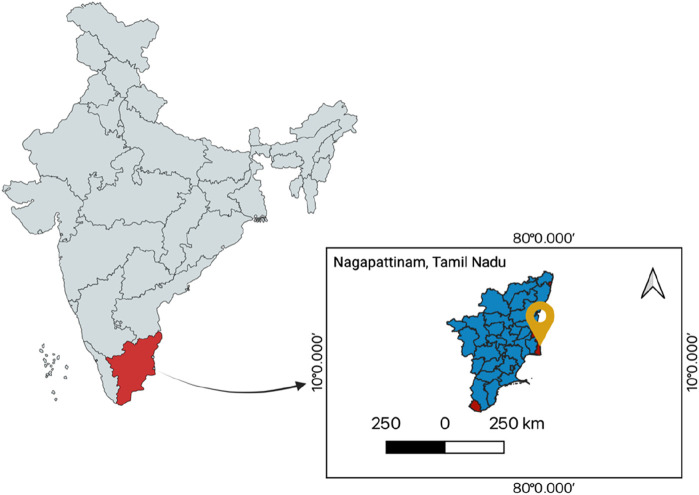
Illustration of the study site in the map (10.7672°N, 79.8449°E) (Created in BioRender. Ray (2025) https://BioRender.com/g90e921).

In the current study, we have analyzed the level of heavy metals bioaccumulation in the muscles, liver, and gills of three fish species (*Nemipterus japonicus, Oreochromis mossambicus,* and *Lates calcarifer*), which were collected from the markets of Nagapattinam, Tamil Nadu, India (Figure 1). Here, we have quantified the concentration of bioaccumulated As, Cd, Cr, Pb, Hg, Sr, and V. Furthermore, to elucidate the potential risk associated with the consumption of contaminated fish, we have conducted a comprehensive health risk assessment considering various parameters: estimated daily intake (EDI), estimated weekly intake (EWI), percentage of provisional tolerable weekly intake (%PTWI), maximum daily intake (MDI), maximum weekly intake (MWI), daily intake level (DIL), limit of consumption rate (CRlim), target hazard quotient (THQ), and cancer risk (CR). Accordingly, the study was designed to evaluate organ-resolved bioaccumulation patterns of selected metal(loid)s in commercially relevant marine fish species from Nagapattinam and to assess potential human exposure and health risk under deterministic consumption assumptions for adult and child populations. While the findings provide useful baseline information on organ-specific bioaccumulation and associated health risk indices, the present dataset is based on a limited number of samples (n = 3 per species) collected during a single seasonal window (January–February 2024). Therefore, the results should be interpreted as exploratory and indicative rather than representative of the broader fish population across seasons. For the overall analysis, we selected an ICP-MS instrumentation facility. The R^2^ value ranges from 0.9981 to 1.00 as per our QC data, demonstrating the efficiency, consistency, and robustness of the method employed.

## 2 Materials and methods

### 2.1 Site description

Our study area, Nagapattinam in the state of Tamil Nadu (TN) (Figure 1), is one of the major harbors in TN. It is located along the Bay of Bengal on the east coast of India. The district is primarily coastal, with a 141-km stretch of coastline, and the port of Nagapattinam plays an essential role in fishing and trading operations occurring in the region. It serves as an important hub for seafood landing, processing, and export activities (Department of Fisheries, 2020).

Due to its coastal location and intensive marine activities, the area is potentially influenced by multiple sources of metal inputs, including harbor operations, boat maintenance activities, fuel combustion, seafood processing units, agricultural runoff from surrounding delta regions, and domestic or municipal effluents entering coastal waters. Therefore, assessment of metal accumulation in commercially important fish species from this harbor environment is relevant for understanding potential exposure risks to consumers.

### 2.2 Sample processing

Three distinct species, specifically *Nemipterus japonicus* (commonly called Rani fish), *Oreochromis mossambicus* (commonly called Jalebi fish), and *Lates calcarifer* (commonly called Koduva fish), were procured from the commercial markets located in Nagapattinam (10.7672°N, 79.8449°E). The fish were dead when purchased and were intended for human consumption (Ray and Vashishth, 2024). A total of nine fish specimens were collected by adopting a purposive sampling technique with three individuals representing each of the three species. Species were selected based on their commercial importance and regular availability in local markets. However, individual specimens within each species were selected randomly from the landing batch to reduce selection bias. The sample size was constrained by logistical and resource considerations and was intended to provide a preliminary, exploratory assessment of organ-specific metal accumulation in the selected commercially important species. The present dataset should therefore be interpreted as indicative rather than statistically representative of the broader fish population across seasons. The sampling period extended from the conclusion of January to February 2024, corresponding to a single seasonal period along the southeast coast of India. The present dataset, therefore, represents a cross-sectional assessment rather than a multi-seasonal evaluation. The mean lengths of *Nemipterus japonicus, Oreochromis mossambicus,* and *Lates calcarifer* were 16.1 ± 0.72 cm, 16.9 ± 0.30 cm, and 15.9 ± 0.24 cm, respectively. The fish specimens were meticulously dissected utilizing a sharp, Teflon knife. Subsequently, the musculature, hepatic tissue, and gill structures were meticulously excised. These isolated organs were then subjected to desiccation in a laboratory oven set at 40 °C until they reached a consistent weight. The desiccated homogenized samples were further processed into a fine powder utilizing a mortar and pestle apparatus. A total of three replicates (n = 3) were designated for subsequent analytical procedures. It should be noted that the present investigation focused exclusively on fish tissues as bioindicators of metal exposure. Environmental media such as water and sediment were not sampled in this study; therefore, the findings reflect biotic accumulation patterns rather than direct measurements of ambient environmental concentrations.

### 2.3 Extraction and analysis

A validated extraction technique derived from a contemporary research investigation was employed to facilitate the preparation of samples for the quantification of heavy metals (HMs) (Griboff et al., 2017). A closed vessel acid digestion employing a heat-resistant receptacle (specifically, Teflon tubes) was utilized for the digestion procedure. A combined volume of 1 mL of Merck 30% hydrogen peroxide ultrapure (H_2_O_2_) and 8 mL of laboratory-grade 67% nitric acid (HNO_3_; Nice Chemicals) was administered to digest each 25 mg (dry weight) of the pre-processed samples. The digestion process was conducted at a temperature of 220 °C on a hot plate for an approximate duration of 8 h. Subsequently, the mineralized samples were transferred into a 10 mL volumetric flask and further brought to a total volume of 10 mL through the addition of 2% HNO_3_. Tenfold dilutions of the prepared sample were executed, and this procedure was replicated for all samples included in the study.

### 2.4 Instrumental setup

The inductively coupled plasma mass spectrometry (ICP-MS) apparatus, PerkinElmer NexION 1000, installed at Vellore Institute of Technology and funded by the Department of Science and Technology, New Delhi, via the Promotion of University Research and Scientific Excellence (PURSE) initiative, was employed for the quantification of metal concentrations (As, Cd, Cr, Hg, Pb, Sr, and V) in aqueous samples. This apparatus was outfitted with a fixed injector torch characterized by an inner diameter of 1.5 mm, a spray chamber thermally regulated by a Peltier device to diminish solvent load through the attenuation of sample aerosol temperature, and a microflow concentric nebulizer for sample introduction. Furthermore, an autosampler of the PerkinElmer S23 model was integrated into the configuration. To enhance sensitivity and mitigate contamination, a pre-installed triple cone interface comprising a sampler cone, a skimmer cone, and a hyper skimmer cone was utilized, with the radio frequency (RF) forward power level calibrated at 1600 W. The operational modality was configured to employ the helium kinetic energy discrimination (KED) mode in the absence of collision cell technology (CCT). Prior to the initiation of Q-ICP-MS analyses, all fish tissue samples underwent a tenfold dilution with 2% HNO_3_, while standards and blanks were formulated utilizing 2% HNO_3_ (Ray and Vashishth, 2024). The isotopes ^75^As, ^111^Cd, ^52^Cr, ^202^Hg, ^208^Pb, ^88^Sr, and ^51^V were monitored for quantification. Helium collision mode (He KED) was applied where necessary to minimize polyatomic interferences. External calibration was performed using multi-element standards prepared in the same acid matrix as samples over an appropriate working range covering expected tissue concentrations. Internal standards were used to correct for instrumental drift and matrix effects. Limits of detection (LOD) and limits of quantification (LOQ) for each element are provided in Section 2.5.

The high R^2^ (coefficient of determination) value range (0.9981–1.00) obtained from the linear regression of all seven elemental concentrations suggests that the calibration standards used in the ICP-MS analysis are robust and provide reliable quantification. In addition, analytical accuracy was evaluated using spiked matrix samples, and the method demonstrated acceptable recoveries for all elements. Analytical precision was confirmed through triplicate measurements of each sample, with relative standard deviation (RSD) values ≤5%, further supporting the reliability and robustness of the analytical procedure.

### 2.5 Quality control and assurance

Analyses of blanks and spiked matrix samples were done for every batch of sample (one blank and one spiked sample for every 10 tissue samples). The limit of detection (LOD) was found to be in the range of 0.001–0.010 μg/kg, and the limit of quantification (LOQ) was found to be in the range of 0.005–0.030 μg/kg, depending on the element. Analytical precision was confirmed by analyzing each sample in triplicate. The relative standard deviation (RSD) for all the analyses was 5%. All concentrations measured in the present study were above the respective LOD values. Therefore, no substitution procedures (e.g., LOD/2 or LOQ/2) were required for statistical analysis or health risk assessment calculations. The triplicate measurements refer to technical replicate analyses of each digested sample, and the averaged values were used for subsequent statistical evaluation. Method accuracy was further evaluated using spiked tissue matrix samples prepared alongside the study samples. Percentage recoveries for the analyzed elements were within acceptable analytical ranges (90%–105%), confirming the reliability of the digestion and quantification procedures. No certified reference material (CRM) for fish tissue was analyzed in the present study.

### 2.6 Health risk assessment

In order to evaluate the potential health hazards (non-carcinogenic) associated with the ingestion of *Nemipterus japonicus*, *Oreochromis mossambicus*, and *Lates calcarifer*, calculations were conducted for a range of parameters (Ray and Vashishth, 2024): estimated daily intake (EDI), estimated weekly intake (EWI), percentage of provisional tolerable weekly intake (%PTWI), maximum daily intake (MDI), maximum weekly intake (MWI), daily intake level (DIL), limit of consumption rate (CRlim), target hazard quotient (THQ), and cancer risk (CR) (FAO/WHO, 2011; Gao et al., 2021; Javed and Usmani, 2019; Naughton and Petróczi, 2008; Scientific Opinion on Lead in Food, 2010; USEPA National Center For Environmental AssessmenT, 2011; USEPA, 1989; 2004; WHO, 2017) (Tables 1, 2). While measuring heavy metal concentrations and comparing them to safety standards is an important step, a risk assessment provides additional critical information that goes beyond simple concentration measurements.

**TABLE 1:** Summary of various variables used in the article, along with symbol, unit, value (if any), and brief description.

Variable symbol	Full name	Value	Unit	Brief description
Mc	Metal concentration in fish	Measured value (varies)	µg/kg	Concentration of the heavy metal in fish muscle
IR	Ingestion rate	75 (children); 150 (adults)	g/day	Rate of fish consumption
BW	Body weight	20 (children); 70 (adults)	kg	Average body weight of individuals
EFr	Exposure frequency	365	Days/year	Frequency of exposure per year (daily exposure assumed)
ED	Exposure duration	70	Years	Lifetime exposure duration
PTWI	Provisional tolerable weekly intake	Varies (based on metal)	µg/kg BW/week	Permissible weekly intake limit for heavy metals (e.g., As: 15, Cd: 7, Pb: 25, Hg: 4, Sr: 7,000, and V: 7)
RfD	Reference dose	Varies (e.g., As: 0.0003)	mg/kg/day	Safe daily intake level for individual metals
CSF	Cancer slope factor	Varies (e.g., Cd: 5 × 10^−5^)	mg/kg/day	Cancer potency factor for carcinogenic metals

**TABLE 2:** Heavy metals and the respective PTWI and RfD values, along with the health effects.

Heavy metal	PTWI	Unit	RfD	Unit	Health effect	References
Arsenic (As)	15	µg/kg body weight per week	0.0003	mg/kg/day	Chronic exposure is linked to skin lesions, cancer, and cardiovascular diseases	Balali-Mood et al. (2021); FAO/WHO (2011); Scientific Opinion on Lead in Food (2010)
Cadmium (Cd)	7		0.001		Kidney damage, bone demineralization, and cancer risks	
Lead (Pb)	25		0.0035		Neurological disorders and developmental delays in children	
Mercury (Hg)	4		0.0001		Neurotoxicity, developmental toxicity, and kidney damage	
Strontium (Sr)	7000		0.6		Bone health effects (high doses may cause bone disorders)	
Vanadium (V)	7		0.009		Respiratory and gastrointestinal effects	
Chromium (Cr III)	1.5		1.5		Essential nutrient at low levels, but high doses may cause gastrointestinal issues	
Chromium (Cr VI)	0.003		0.003		Carcinogenic effects and respiratory issues	

The Equation 1–8 used for the assessment are presented below.

$$\text{EDI}=\left(\text{Mc }x\text{ IR}\right)/\text{body weight}.$$ (1)

Here, Mc is the amount of metal in fish muscle, IR is the rate of consumption (considered values: 75 g/day for children and 150 g/day for adults), and BW is body weight (considered values: adult 70 kg; children under the age of 7 years were considered to be 20 kg) (USEPA, 2004).

The ingestion rates (IR) of 75 g day^−1^ for children and 150 g day^−1^ for adults were adopted from commonly applied default consumption scenarios in fish-related human health risk assessments (USEPA, 2011; Javed and Usmani, 2016; Noman et al., 2022). These values represent generalized exposure assumptions rather than site-specific dietary survey data for the Nagapattinam population. In the absence of detailed local seafood consumption statistics, these standardized intake rates were applied to facilitate comparison with previous studies and regulatory frameworks.

$$\text{EWI}=\left[\left(\text{Mc }x\text{ IR}\right)/\text{BW}\right]x\;7,$$ (2)

$$\%PTWI=\left(\frac{Actual\;weekly\;intake}{PTWI}\right)\times 100.$$ (3)

PTWI values for arsenic (inorganic), Cd, Pb, Hg, Sr, and V are 15 µg/kg, 7 µg/kg, 25 µg/kg, 4 µg/kg, 7000 µg/kg, and 7 μg/kg body weight per week, respectively (Scientific Opinion on Lead in Food, 2010).

$$MDI=\frac{\left(PTWI\times Body\;Weight\right)}{7},$$ (4)

$$DIL=\frac{\;RfD\times BW}{C},$$ (5)

$$CRlim=\frac{\text{RfD}\times \text{BW}}{C}.$$ (6)

Here, the RfD is the reference dose of the heavy metal [As, Cd, Cr(III), Cr(VI), Hg, Pb, Sr, and V are 0.0003 mg/kg/day, 0.001 mg/kg/day, 1.5 mg/kg/day, 0.003 mg/kg/day, 0.0001 mg/kg/day, 0.0003 mg/kg/day, 0.0035 mg/kg/day, 0.6 mg/kg/day, and 0.009 mg/kg/day, respectively)] (FAO/WHO, 2011; WHO, 2017). CRlim or MADI is the estimate of the daily exposure to the human population (including sensitive subgroups) that is likely to be without an appreciable risk of harmful effects during a lifetime.

$$THQ=\frac{\text{EFr}\times \text{ED}\times \text{IR}\times C}{RfD\times BW\times AD},$$ (7)

where EFr is exposure frequency (365 days/year is the default for daily exposure) and ED is exposure duration (70 years for a lifetime exposure) (Naughton and Petróczi, 2008).

CR=EDI×CSF. (8)

The cancer slope factor (CSF) values for Cd, Pb, Cr, and As are 5 × 10^−5^ mg/kg/day, 8.5 × 10^−3^ mg/kg/day, 41 mg/kg/day, and 1.5 mg/kg/day (FAO/WHO, 2011; Gao et al., 2021), respectively.

Chromium concentrations quantified by ICP-MS represent total chromium, as elemental analysis was performed without a prior speciation. Because ICP-MS does not differentiate between trivalent chromium [Cr(III)] and hexavalent chromium [Cr(VI)], carcinogenic risk (CR) for chromium was estimated using the oral slope factor established for Cr(VI) according to USEPA risk assessment guidelines (USEPA, 1989; USEPA, 2004). This approach represents a conservative worst-case assumption in which total chromium is considered equivalent to Cr(VI). It is therefore acknowledged that the calculated CR values for chromium may overestimate the actual carcinogenic risk.

### 2.7 Statistical analysis

The experiments were conducted in triplicate, with the resulting data expressed as mean ± standard deviation (SD). For each metal, we tested for differences among species and organs using two-way analysis of variance (ANOVA) with interaction (factors: species × organ). Model residuals were evaluated for normality using the Shapiro–Wilk test and for homogeneity of variances using Levene’s test. Where assumptions were not met, data were log-transformed [log (x + small constant)], and the ANOVA was rerun on transformed data. Pairwise group comparisons were performed with Tukey’s honestly significant difference (HSD). Effect sizes (η^2^) and assumptions test results are reported alongside ANOVA tables.

## 3 Results and discussion

### 3.1 Heavy metal estimation

The study found that As, Cd, Cr, Hg, Pb, Sr, and V occurred in trace concentrations of 0.025–0.44 μg kg^−1^, 0.65–0.77 μg kg^−1^, 4.36–10.69 μg kg^−1^, 0.48–2.32 μg kg^−1^, 14.53–17.10 μg kg^−1^, 0.59–21.53 μg kg^−1^, and 0.14–0.55 μg kg^−1^, respectively, in the different organs of the three selected species: *Nemipterus japonicus*, *Oreochromis mossambicus*, and *Lates calcarifer* (Table 3). The concentration of HMs varied significantly across the organs (p < 0.05). The findings of this study are based on a limited sample size and do not account for temporal, spatial, or biological variations. Future studies will incorporate larger sample sizes, multiple sampling seasons, and consideration of factors such as habitat, size, and gender differences.

**TABLE 3:** Heavy metal concentrations in liver, gills, and muscle of three selected species from Nagapattinam, TN (in μg kg^−1^) (represented as mean ± standard deviation; n = 3).

Fish	Organ	As	Cd	Cr	Hg	Pb	Sr	V
*Nemipterus japonicus*	Liver	0.063 ± 0.01	0.680 ± 0.07	4.43 ± 0.61	0.85 ± 0.63	14.83 ± 1.7	1.17 ± 0.47	0.16 ± 0.07
	Gills	0.034 ± 0.01	0.662 ± 0.02	4.71 ± 0.34	0.48 ± 0.26	14.59 ± 0.41	0.63 ± 0.21	0.14 ± 0.009
	Muscle	0.436 ± 0.04	0.674 ± 0.04	10.69 ± 3.13	1.46 ± 1.67	15.21 ± 1.19	5.16 ± 0.72	0.21 ± 0.04
*Oreochromis mossambicus*	Liver	0.099 ± 0.006	0.777 ± 0.02	9.16 ± 0.46	0.71 ± 0.81	17.10 ± 0.70	14.25 ± 1.19	0.55 ± 0.03
	Gills	0.032 ± 0.01	0.676 ± 0.02	4.54 ± 0.15	0.61 ± 0.65	14.53 ± 0.2	0.64 ± 0.23	0.15 ± 0.01
	Muscle	0.230 ± 0.25	0.727 ± 0.12	10.07 ± 5.52	0.74 ± 0.79	14.60 ± 0.7	7.15 ± 6.73	0.27 ± 0.12
*Lates calcarifer*	Liver	0.444 ± 0.07	0.719 ± 0.03	8.30 ± 0.72	0.54 ± 0.53	15.01 ± 0.46	3.12 ± 1.13	0.21 ± 0.02
	Gills	0.443 ± 0.11	0.702 ± 0.05	8.29 ± 1.72	2.32 ± 2.73	15.79 ± 1.9	21.53 ± 5.03	0.38 ± 0.09
	Muscle	0.025 ± 0.001	0.650 ± 0.01	4.36 ± 0.11	1.01 ± 0.98	14.53 ± 0.49	0.59 ± 0.15	0.15 ± 0.01

The peak concentration of As was identified in the hepatic tissue of *Lates calcarifer* (0.444 μg/kg), whereas the muscular tissue of *Lates calcarifer* exhibited the lowest As concentration (0.025 μg/kg). Generally, muscle tissues are characterized by lower As concentrations, which is advantageous because muscle constitutes the primary portion consumed by humans (Griboff et al., 2017b; Javed and Usmani, 2016; 2019; Noman et al., 2022). Conversely, the liver exhibited a more pronounced accumulation of As, potentially indicating increased exposure to environmental contaminants. Cd levels demonstrated considerable consistency across various species, with the hepatic tissue of *Oreochromis mossambicus* presenting the highest concentration (0.777 μg/kg). The Cd concentration in muscle was found to be lower, thereby diminishing the likelihood of human exposure. Cd is recognized for its deleterious effects on renal and skeletal systems, making its accumulation in consumable muscle tissue a significant health concern. Cr was found at the highest concentration among the metals examined, particularly within the muscle of *Nemipterus japonicus* (10.69 μg/kg). The liver and gills also exhibited elevated Cr concentrations, especially in *Lates calcarifer*. However, the muscle of *Lates calcarifer* revealed relatively modest Cr levels (4.36 μg/kg). While Cr(III) is acknowledged as an essential nutrient, elevated levels of Cr, particularly Cr(VI), present serious health risks, including carcinogenic potential. Hg concentrations exhibited variability, with the highest levels recorded in the gills of *Lates calcarifer* (2.32 μg/kg). The presence of Hg in muscle tissue (1.46 μg kg^−1^ in *Nemipterus japonicus*) does not exceed established regulatory limits but warrants consideration in exposure assessment, particularly under higher consumption scenarios, as Hg can induce neurological damage and bioaccumulates within biological organisms (Balali-Mood et al., 2021; Barone et al., 2015). The comparatively lower Hg concentrations in muscle tissues across species, as opposed to the liver and gills, represent a favorable outcome for consumer safety. Pb concentrations were particularly elevated across species, with *Oreochromis mossambicus* demonstrating heightened levels in both hepatic (17.10 μg/kg) and muscular (14.60 μg/kg) tissues. This situation is critical due to the potential for Pb exposure to induce developmental and neurological issues in humans (Balali-Mood et al., 2021). The muscular tissues of all three species contained Pb concentrations at or above 14 μg/kg, which could raise concerns from a public health standpoint, particularly for children and other vulnerable populations. Sr concentrations were markedly high in the gills of *Lates calcarifer* (21.53 μg/kg), indicating its substantial environmental availability. Strontium is infrequently monitored within food safety assessments; however, elevated concentrations could pose a concern for bone health if ingested in substantial quantities (MARIE, 2005; Ru et al., 2024; Wnuk, 2023). Vanadium/V levels were comparatively low relative to other metals, with the liver of *Oreochromis mossambicus* exhibiting the highest concentration (0.55 μg/kg). Although V is regarded as less toxic than several other heavy metals, chronic exposure may still present health risks, particularly in aquatic organisms (Wnuk, 2023).

**FIGURE 2:**
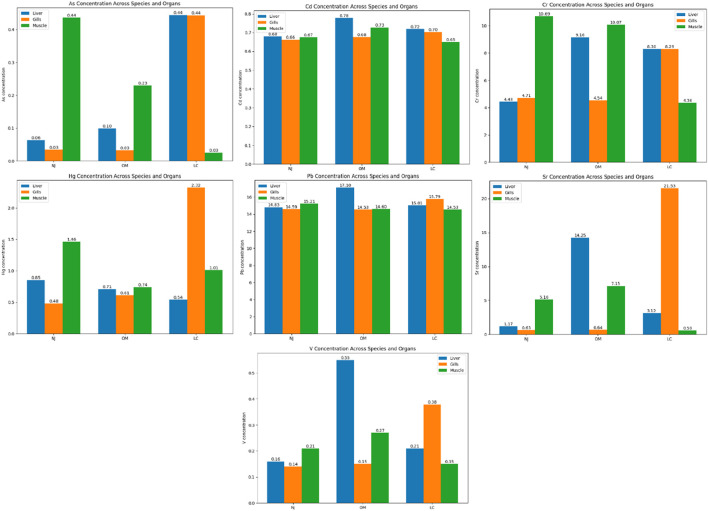
Graphs representing the heavy metal contamination (in ppb) in three different organs of selected fish species. The fish species were marked as NJ, OM, and LC for *Nemipterus japonicus, Oreochromis mossambicus*, and *Lates calcarifer*, respectively.

*Oreochromis mossambicus* had the highest levels of Pb, making it a species of concern for human consumption (Figure 2). *Nemipterus japonicus* showed significant accumulation of Cr and Hg, underscoring the need to monitor these metals for consumer safety (Figure 2). *Lates calcarifer* demonstrated a mixed profile, with high Hg and Sr concentrations in the gills but lower levels in muscle, which is beneficial for consumption safety. Overall, the contamination level in the muscle of *Nemipterus japonicus* was found to be in the order of Pb > Cr > Sr > Cd > Hg > V > As. For *Oreochromis mossambicus* and *Lates calcarifer*, the contamination levels in the muscle were found to be Pb > Cr > Sr > Cd > Hg > V > As and Pb > Sr > Cr > Hg > Cd > V > As, respectively.

A comparable investigation carried out in 2021 within the Gulf of Guinea by Botwe, who documented the presence of Hg contamination in *D. angolensis* (0.14 ± 0.03 μg/kg) (Botwe, 2021). The Hg concentrations identified in our samples were comparatively higher than those reported by Botwe (2021), and all values are expressed in µg/kg to ensure direct comparison. However, the Hg contamination level found in our study does not pose a significant potential non-carcinogenic risk under the assumed consumption scenario.

The findings were also compared with the research performed at Ennore Creek (Tamil Nadu, India) in 2013 by Kumar C., wherein the levels of lead contamination were reported in µg/g (Kumar et al., 2013). The Pb concentrations in *Penaeus monodon*, *Perna viridis*, *Crassostrea madrasensis*, *Mugil cephalus*, and *Terapon jarbua* were assessed at 4.37 ± 0.33 µg/g, 3.42 ± 0.29 µg/g, 4.00 ± 0.29 µg/g, 2.59 ± 0.31 µg/g, and 3.42 ± 0.29 μg/g, respectively (Kumar et al., 2013), which correspond to 2,590–4,370 μg/kg after unit conversion (1 μg/g = 1,000 μg/kg). In comparison, the Pb contamination levels recorded in the present study are substantially lower than those in earlier reports, indicating comparatively reduced Pb contamination in the studied region.

The liver consistently demonstrated elevated concentrations of various metals across all species. This phenomenon is anticipated, given that the liver functions as an important organ in the detoxification processes of heavy metals, effectively sequestering and metabolizing deleterious substances. Gills were found to have considerable amounts of various metals, particularly mercury (Hg), chromium (Cr), and strontium (Sr). Although muscle tissues typically display lower metal concentrations, certain exceptions are noteworthy, specifically concerning lead (Pb) and chromium (Cr) in the particular species in our study (Figure 2). Considering that muscle constitutes the primary tissue ingested by humans, the presence of these metals warrants evaluation against regulatory standards. In the present study, muscle Pb concentrations (∼14–17 μg kg^−1^) and Hg concentrations (∼0.7–1.5 μg kg^−1^) are substantially below the maximum limits established by international guidelines (e.g., Pb: 0.3 mg kg^−1^ wet weight; Hg: 0.5 mg kg^−1^ for most fish species). Therefore, the observed levels do not exceed current regulatory thresholds. However, prolonged consumption or higher intake rates, particularly among children or high seafood-consuming populations, may proportionally increase exposure and should be considered in risk interpretation.

Numerous factors may influence the accumulation processes of such contaminants (Anwarul Hasan et al., 2022; El-Moselhy et al., 2014; Ture et al., 2021; Zaghloul et al., 2024). Muscle tissues exhibit reduced exposure to detoxification mechanisms relative to the liver and gills, as these organs are more actively engaged in metabolic processes. Consequently, prolonged accumulation may manifest as elevated concentrations within muscle tissues for this reason (Anwarul Hasan et al., 2022). The composition of fish muscle can vary significantly concerning lipid content, binding site localization, and other factors (Balali-Mood et al., 2021). An elevated lipid content is correlated with enhanced binding efficiency of heavy metals within these tissues (Balali-Mood et al., 2021). The rate at which detoxification or excretion of these contaminants occurs can differ both within individual species and among different species. This biological process impacts the levels of bioaccumulation as well.

### 3.2 Health risk assessment

Our assessment of the estimated daily intake (EDI) values (Table 4), which represents the daily intake of heavy metals through fish consumption, indicates potential health risks over a long period of consumption, suggesting various critical aspects to the overall study (FAO/WHO, 2011). EDI values for As were 0.05–1.64 μg/kg/day across all three selected species. Such a comparatively low range indicated that while As was present in the fish, the risk of acute toxicity was limited. However, frequent and continuous consumption, especially by children, may have a possible chronic health risk (Carroquino et al., 2013). The EDI values for Cd were 1.39–2.72 μg/kg/day, indicating that cadmium levels were relatively similar across the selected species, with moderate levels of contamination in the present study. Because Cd is a cumulative toxin, even these lower daily intakes may cause harmful effects over time, particularly toward vulnerable populations like children. In the case of Cr, the EDI values ranged from 9.34 µg/kg/day to 40.09 μg/kg/day. This wider range, compared to other metals, indicated that some species, such as *Nemipterus japonicus*, bioaccumulated significantly more Cr than other selected species. While Cr is considered to be essential in trace amounts, higher EDI values, especially for Cr(VI), may raise concerns regarding carcinogenicity and other forms of toxicity (Al osman et al., 2019; Ali et al., 2019; Balali-Mood et al., 2021; Bera et al., 2022; Garai et al., 2021; Jaishankar et al., 2014; Jamil Emon et al., 2023). For Hg, the EDI values were 1.58–5.48 μg/kg/day, suggesting moderate to high levels of Hg contamination, particularly in *Nemipterus japonicus*. Hg is considered concerning due to its neurotoxic effects, especially in children (Carocci et al., 2014). Even at the lower end of this range, Hg exposure through fish consumption requires careful monitoring. Furthermore, the EDI values for Pb were 31.13–57.04 μg/kg/day, indicating high Pb levels across the selected species, especially for children. The EDI values for Sr were 1.26–26.81 μg/kg/day, suggesting a variability in Sr accumulation across species, with higher levels observed in *Oreochromis mossambicus*. Although Sr is less toxic than other metals, its presence at higher levels could have contributed to cumulative toxicity over time. In case of V, the EDI values were 0.32–1.01 μg/kg/day, indicating relatively low V contamination across all fish species. This suggested minimal risk from daily exposure to V, though continuous consumption over time may lead to accumulation and potential adverse health effects. The EWI values followed similar patterns to the EDI values, reinforcing the previous concerns about daily intake.

**TABLE 4:** Assessment: EDI, EWI, DIL, and CRlim data (RfD values of As, Cd, Cr(III), Cr(VI), Hg, Pb, Sr, and V are 0.0003 mg/kg/day, 0.001 mg/kg/day, 1.5 mg/kg/day, 0.003 mg/kg/day, 0.0001 mg/kg/day, 0.0003 mg/kg/day, 0.0035 mg/kg/day, 0.6 mg/kg/day, and 0.009 mg/kg/day, respectively) (‘-’ is not determined).

Fish species	​	Estimated daily intake (EDI) in µg/kg/day		Estimated weekly intake (EWI) in µg/kg/day		Daily intake limit for fish in kg/day		CRlim/maximum acceptable daily intake (kg/day)	
​	Heavy metals	Children	Adult	Children	Adult	Children	Adult	Children	Adult
*Nemipterus japonicus*	As	1.64	0.93	11.48	6.51	13.76	48.16	13,761.46	48,165.13
	Cd	2.53	1.44	17.71	10.08	29.67	103.85	29,673.59	103,857.56
	Cr	40.09	22.91	280.63	160.37	2806.36 (Cr VI)	9822.26 (Cr VI)	2,806,361.08	9,822,263.79
	Hg	5.48	3.13	38.36	21.91	1.36	4.79	1369.86	4794.52
	Pb	57.04	32.59	399.28	228.13	0.39	1.38	394.48	1380.67
	Sr	19.35	11.06	135.45	77.42	13.56	47.48	13,565.89	47,480.62
	V	0.79	0.45	5.53	3.15	857.14	3000	857,142.86	3,000,000.00
*Oreochromis mossambicus*	As	0.86	0.49	6.02	3.43	26.08	91.3	26,086.96	91,304.35
	Cd	2.72	1.55	19.04	10.85	27.51	96.28	27,510.32	96,286.11
	Cr	37.76	21.57	264.32	150.99	2979.14 (Cr VI)	10,427.01 (Cr VI)	2,979,145.98	10,427,010.92
	Hg	2.77	1.58	19.39	11.06	2.7	9.45	2702.70	9459.46
	Pb	54.75	31.28	383.25	218.96	0.41	1.43	410.96	1438.36
	Sr	26.81	15.32	187.67	107.24	9.79	34.26	9790.21	34,265.73
	V	1.01	0.57	7.07	3.99	666.66	2333.33	666,666.67	2,333,333.33
*Lates calcarifer*	As	0.09	0.05	0.63	0.35	240	840	240,000.00	840,000.00
	Cd	2.43	1.39	17.01	9.73	30.76	107.69	30,769.23	107,692.31
	Cr	16.35	9.34	114.45	65.38	6880.73 (Cr VI)	24,082.56 (Cr VI)	6,880,733.94	24,082,568.81
	Hg	3.78	2.16	26.46	15.12	1.98	6.93	1980.20	6930.69
	Pb	54.48	31.13	381.36	217.91	0.41	1.44	412.94	1445.29
	Sr	2.21	1.26	15.47	8.82	118.64	415.25	118,644.07	415,254.24
	V	0.56	0.32	3.92	2.24	1200	4200	1,200,000.00	4,200,000.00

In terms of the daily intake limit (DIL), the values indicated here are the maximum safe quantity of fish that can be consumed daily without exceeding the toxic thresholds provided by the regulating authority. Lower DIL values indicated higher risks associated with fish consumption. In the case of As, the DIL values were 13.76–840 kg/day across all the species, suggesting that As posed a relatively low risk at the detected levels. This allowed for a relatively high daily intake of fish before reaching unsafe As exposure levels. The DIL values for Cd, 27.51–107.69 kg/day, indicate moderate Cd contamination across species. For Cr(VI), the DIL values ranged from 2806.36 kg/day to 10,427.01 kg/day, reflecting the fact that the Cr contamination was negligible, and the risk of toxicity remained relatively very low unless the fish consumption exceeded more than one person could consume. On the other hand, for Hg, the DIL values are 1.36–9.45 kg/day. Hg poses a much higher risk than other metals, as indicated by these low DIL values, but the risk still remains under control. The DIL values for Pb, 0.39–1.44 kg/day, are the lowest among the metals in our study, indicating that even small amounts of fish consumption may lead to harmful impacts. Furthermore, for Sr and V, the DIL values were 9.79–415.25 kg/day and 666.66–4200 kg/day, with V showing the highest DIL values in our study, reflecting its relatively low or negligible toxicity in most cases and minimal health risk association at the detected levels.

Table 5 provides the information about maximum daily and weekly intake limits of As, Cd, Cr, Hg, Pb, Sr, and V for children and adults. As and Cd were reported to have maximum daily intake limits of 0.043 mg/day and 0.02 mg/day for children and 0.15 mg/day and 0.07 mg/day for adults, with weekly limits of 0.301 mg/week and 0.14 mg/week for children, and 1.05 mg/week and 0.49 mg/week for adults, respectively. This further highlighted its toxicity even at low levels, especially for children, suggesting its potential for cumulative or combined toxicity, particularly for the renal tissues and associated organs. Hg and Pb, with daily intakes of 0.07 mg/day and 0.01 mg/day for children, and weekly intakes of 0.49 mg/week and 0.04 mg/week for adults, respectively, emphasizing the possible risks associated with lead exposure. Sr and V, while being a concern, have higher permissible intake levels. Sr was reported to range up to 20 mg/day for children and 70 mg/day for adults, and the V range was up to 0.02 mg/day for children and 0.07 mg/day for adults, indicating relatively lower toxicity than the other metals.

**TABLE 5:** MDI, MWI [PTWI for arsenic (inorganic), Cd, Pb, Hg, Sr, and V are 15 µg/kg, 7 µg/kg, 25 µg/kg, 4 µg/kg, 7000 µg/kg, and 7 μg/kg body weight per week] (In the table all values are regulatory value).

Heavy metals	Maximum daily intake (in mg/day)		Maximum weekly intake (mg/week)	
​	Children	Adult	Children	Adult
As	0.043	0.15	0.301	1.05
Cd	0.02	0.07	0.14	0.49
Cr	_	_	_	_
Hg	0.07	0.25	0.49	1.75
Pb	0.01	0.04	0.07	0.28
Sr	20	70	140	490
V	0.02	0.07	0.14	0.49

An assessment of the target hazard quotient (THQ) was conducted (Table 6). In all the cases for the three selected species, the THQ <1 suggests low individual non-carcinogenic risk; however, mixture effects and unprofiled contaminants warrant caution. The cancer risk assessment indicated a lower risk of cancer overall. Regulatory bodies like the USEPA consider a cancer risk of 1 × 10^−6^ to be acceptable (USEPA, 1989; 2004). Here, the values are expressed as a factor of 10^–6^ (a risk of 1 × 10^−6^ refers to a 1-in-a-million chance of developing cancer from lifetime exposure to the given substance). Sr and V are not typically classified as carcinogens, and hence, there is no clear cancer slope factor (CSF) for such metals.

**TABLE 6:** Assessment: %PTWI, THQ, and CR (‘-’ is not determined).

Fish species	​	Percentage of provisional tolerable weekly intake (%PTW)		Target hazard quotient (THQ)		Cancer risk	
	Heavy metals	Children	Adult	Children	Adult	Children	Adult
*Nemipterus japonicus*	As	76.3	43.6	0.0055	0.0031	2.45 × 10^−6^	1.40 × 10^−6^
	Cd	252.75	144.42	0.0025	0.0014	0.0001 × 10^−6^	0.0001 × 10^−6^
	Cr	28,061.25	16,035	0.0000	0.0000	1643.58 × 10^−6^	939.19 × 10^−6^
	Hg	958.12	547.5	0.0548	0.0313	_	_
	Pb	1597.05	912.59	0.1901	0.1086	0.4848 × 10^−6^	0.2770 × 10^−6^
	Sr	1.93	1.1	0.0055	0.0032	_	_
	V	78.75	45	0.0001	0.0001	_	_
*Oreochromis mossambicus*	As	40.25	23	0.0029	0.0016	1.29 × 10^−6^	0.73 × 10^−6^
	Cd	272.62	155.78	0.0027	0.0016	0.0001 × 10^−6^	0.0001 × 10^−6^
	Cr	26,433.75	15,105	0.0000	0.0000	1548.26 × 10^−6^	884.72 × 10^−6^
	Hg	485.62	277.5	0.0278	0.0159	_	_
	Pb	1533	876	0.1825	0.1043	0.46 × 10^−6^	0.26 × 10^−6^
	Sr	2.68	1.53	0.0077	0.0044	_	_
	V	101.25	57.85	0.0001	0.0001	_	_
*Lates calcarifer*	As	4.37	2.5	0.0003	0.0002	0.140 × 10^−6^	0.08 × 10^−6^
	Cd	243.75	139.28	0.0024	0.0014	0.0001 × 10^−6^	0.0001 × 10^−6^
	Cr	11,445	6540	0.0000	0.0000	670.35 × 10^−6^	383.05 × 10^−6^
	Hg	662.81	378.74	0.0379	0.0216	_	_
	Pb	1525.64	871.8	0.1816	0.1038	0.46 × 10^−6^	0.26 × 10^−6^
	Sr	0.22	0.12	0.0006	0.0004	_	_
	V	56.25	32.14	0.0001	0.0000	_	_

CR values for As ranged from 2.45 × 10^−6^ (*Nemipterus japonicus*) to 0.140 × 10^−6^ (*Lates calcarifer*) in children and from 1.40 × 10^−6^ (*N. japonicus*) to 0.08 × 10^−6^ (*Lates calcarifer*) for adults. As, which is a well-known carcinogen and reported in various studies for its carcinogenic activities, has a well-established cancer slope factor (CSF). Although the CR values here were relatively low, the upper range of the values exceeds the commonly accepted risk threshold of 1 × 10^−6^, indicating that the consumption of the concerned fish species, particularly in children, may pose a marginal risk of cancer due to As exposure. However, the values are still below levels of significant risk that would trigger the recommendation of public health actions. The arsenic concentrations reported in the present study represent total arsenic (As_total), as speciation between inorganic and organic forms was not performed. It should be noted that carcinogenic risk benchmarks and PTWI values are generally based on inorganic arsenic (As_inorganic), which is the more toxic fraction. Therefore, estimating cancer risk using total arsenic concentrations may lead to over- or underestimation, depending on the actual proportion of inorganic arsenic present in the fish tissues.

CR values considering Cd for all fish species for both children and adults were reported as 0.0001 × 10^−6^. These values are extremely low and nearly negligible. Cd has a low potential to cause cancer compared to other metals, especially when considering dietary exposure rather than inhalation (the primary route of concern for cadmium-related cancer risk) (Balali-Mood et al., 2021). This suggests minimal cancer risk from cadmium exposure through the consumption of certain fish.

Chromium exists in two forms, namely Cr(III), which is an essential nutrient, and Cr(VI), which is highly carcinogenic. It is important to note that ICP-MS quantifies total chromium and does not distinguish between trivalent [Cr(III)] and hexavalent [Cr(VI)] species. Cr(III) is an essential trace element with comparatively low toxicity, whereas Cr(VI) is classified as carcinogenic and exhibits significantly higher toxicity (Jaishankar et al., 2014; Balali-Mood et al., 2021). Therefore, the CR values estimated in this study assume a conservative worst-case exposure scenario in which total chromium is considered as Cr(VI), potentially inflating cancer risk estimates. CR values for Cr are 1643.58 × 10^−6^ (*Nemipterus japonicus*), 1548.26 × 10^−6^ (*Oreochromis mossambicus*), and 670.35 × 10^−6^ (*Lates calcarifer*) for children. In case of adults, the values are 939.19 × 10^−6^ (*Nemipterus japonicus*), 884.72 × 10^−6^ (*Oreochromis mossambicus*), and 383.05 × 10^−6^ (*Lates calcarifer*). These theoretical values were significant and concerning, particularly in children. The CR values for Cr significantly exceeded the typical acceptable risk threshold of 1 × 10^−6^, indicating a potential health risk. The results obtained include overall Cr values, with no specified values of Cr(VI). Hence, a claim against acute toxicity needs further specified estimation of Cr(VI) levels.

CR values for Pb ranged from 0.4848 × 10^−6^ (*Nemipterus japonicus*) to 0.46 × 10^−6^ (*Lates calcarifer*) for children and from 0.2770 × 10^−6^ (*Nemipterus japonicus*) to 0.26 × 10^−6^ (*Lates calcarifer*) for adults. Lead has no known safe exposure limit for children due to its developmental toxicity at trace levels. Although the cancer risk values here were well below the 1 × 10^−6^ threshold, it is important to note that Pb is more commonly associated with non-carcinogenic developmental effects rather than cancer.

### 3.3 Statistical analysis

For each metal, data were analyzed using two-way ANOVA with factors “species” and “organ,” including their interaction. Before analysis, the normality of residuals (Shapiro–Wilk test) and homogeneity of variances (Levene’s test) were evaluated; where assumptions were violated, data were log (x + small constant) transformed, and the ANOVA was performed again. All the p-values reported are from the final models.

For As, Sr, and V, significant main effects of Species were observed (p < 0.01), while for the remaining metals, the Species effect was not significant (p > 0.05). Organ effects were significant only for V (p < 0.01), whereas for the other metals, Organ effects were not statistically significant (p > 0.05).

Significant species × organ interactions were detected for As, Cr, Sr, and V (p < 0.01). For Hg, Cd, and Pb, the interaction term was non-significant (p > 0.05).

Tukey’s HSD was used to compare pairwise differences across species–organ groups. In general, the liver exhibited the highest concentrations for Cd and Pb, while muscle exhibited the highest concentrations for Cr and Hg, and gills exhibited the highest concentrations for Sr (p < 0.05 where significant). *O. mossambicus* showed the highest mean levels of Cd and Pb in the liver compared with the other species, while *N. japonicus* had higher Cr levels in muscle. Hg concentrations did not differ significantly among species. Sr levels were highest in the gills of all three species and differed significantly among species, depending on organ (p < 0.05 where significant).

Partial η^2^ values indicated that interaction effects explained substantial variance for As, Cr, Sr, and V (η^2^_interaction_, 0.51–0.73), while main effects were generally smaller (η^2^_species_, 0.03–0.18; η^2^_organ_, 0.02–0.13) (Table 7).

**TABLE 7:** Two-way ANOVA summary table.

Metal	Effect	F (df1, df2)	p-value	η^2^
Hg	Species	F (2,18) = 0.54	0.590	0.046
	Organ	F (2,18) = 0.33	0.727	0.028
	Species × organ	F (4,18) = 0.92	0.476	0.157
As	Species	F (2,18) = 8.14	0.003	0.151
	Organ	F (2,18) = 0.86	0.438	0.016
	Species × organ	F (4,18) = 17.88	<0.001	0.665
Cd	Species	F (2,18) = 2.08	0.154	0.142
	Organ	F (2,18) = 1.74	0.204	0.118
	Species × organ	F (4,18) = 0.93	0.470	0.126
Cr	Species	F (2,18) = 0.83	0.451	0.032
	Organ	F (2,18) = 2.92	0.079	0.113
	Species × organ	F (4,18) = 6.56	0.002	0.507
Pb	Species	F (2,18) = 0.57	0.574	0.034
	Organ	F (2,18) = 1.66	0.218	0.100
	Species × organ	F (4,18) = 2.71	0.063	0.326
Sr	Species	F (2,18) = 11.55	<0.001	0.133
	Organ	F (2,18) = 2.99	0.075	0.034
	Species × organ	F (4,18) = 31.80	<0.001	0.730
V	Species	F (2,18) = 11.92	<0.001	0.178
	Organ	F (2,18) = 8.92	0.002	0.133
	Species × organ	F (4,18) = 18.57	<0.001	0.554

The significant species × organ interactions observed for As, Cr, Sr, and V indicate that tissue-specific accumulation patterns differed among species rather than following a uniform trend. For instance, Cr concentrations were markedly elevated in the muscle of *N. japonicus* and *O. mossambicus* compared to *L. calcarifer*, whereas Sr showed disproportionately higher accumulation in the gills of *L. calcarifer*. Similarly, As and V demonstrated species-dependent organ distribution, with relatively higher concentrations in liver tissues for certain species. These interaction patterns suggest that metal uptake and storage are influenced by species-specific physiological and ecological characteristics, which may in turn affect exposure relevance for human consumption, particularly when muscle tissue is considered. Given the limited biological replication (n = 3 per species × organ), the ANOVA results should be interpreted as exploratory. While the analysis detects patterns in organ-specific and species-dependent accumulation, the small sample size limits statistical power; therefore, the findings indicate trends that warrant validation through studies with larger sample sizes.

For most metals, the standard sequence of liver > gill > muscle corresponds with the established knowledge that the liver serves as the primary site for xenobiotic detoxification and trace metal storage. Metals like Cd, Pb, and As can be sequestered and complexed more easily due to their high metabolic activity and metallothionein synthesis (Kawser Ahmed et al., 2016; MNH et al., 2017; Rashed, 2001). Higher hepatic concentrations of Cd and Pb have also been reported in a number of studies on *Oreochromis mossambicus* and *Lates calcarifer* from contaminated coastal environments. These results show the association of biliary excretion pathways and metal-binding proteins (Kawser Ahmed et al., 2016).

Waterborne elements like Cr and V tend to form labile ionic species that can be diffusively absorbed. The primary point of contact with the aquatic environment, the gills, frequently show moderate metal accumulation (Ali et al., 2019; Ali and Khan, 2019; Balali-Mood et al., 2021; Jamil Emon et al., 2023; Velusamy et al., 2014). The increased gill accumulation of Cr and V in *N. japonicus* here is consistent with previous reports that demersal species exhibit higher gill burdens of redox-active metals because of their proximity to sediment (Copat et al., 2013). Although muscle is still essential for determining human dietary exposure to metals, it consistently displayed the lowest concentrations due to its low metabolic turnover and poor binding affinity.

Marked interspecific variation, especially for Pb and Cd (η^2^_species_, 0.03–0.18), indicates that species ecology and feeding behavior play important roles in metal uptake. *O. mossambicus*, a benthopelagic omnivore, likely accumulates more Cd and Pb through sediment interaction and ingestion of detrital matter; these pathways are corroborated by earlier estuarine bioaccumulation studies (Ahmed et al., 2013; Balali-Mood et al., 2021; Boskabady et al., 2018). On the other hand, comparatively lower concentrations in the more pelagic predator *L. calcarifer* point to trophic and dietary segregation that affects the pathways through which the fish accumulates the metals. Variations in metabolic control and detoxification efficacy, such as species-specific differences in metallothionein expression or hepatic enzyme activity, may also contribute to this variation. Particularly when comparing filter-feeding and carnivorous taxa, similar species-driven differences have been observed in tropical and subtropical fish populations (Celik, 2024; Jamil Emon et al., 2023; Javed and Usmani, 2013; Li et al., 2021; Wnuk, 2023). Comparing filter-feeding and carnivorous taxa in tropical and subtropical fish populations has revealed similar species-driven differences (Kumar et al., 2013; Rubalingeswari et al., 2021; Thiyagarajan et al., 2012).

The lack of significant interspecific variation for Sr, coupled with its strong organ-level effect, suggests that Sr distribution is governed more by physiological regulation than by ecological factors. Instead of being species-dependent, strontium tends to accumulate preferentially in calcified or osmoregulatory tissues like gills and bones because it is chemically similar to calcium (Baatrup, 1991; Kumar et al., 2026). All species have consistently higher gill Sr levels, which support their ionic mimicry of Ca^2+^ and allow substitution during gill membrane ion exchange processes. However, the significance of the interaction term (species × organ) for elements like As, Cd, Pb, and Cr highlights the non-uniform bioaccumulation patterns, which may be due to complex metal–protein binding kinetics, habitat-specific exposure gradients, or trophic interactions. As observed in several marine bioindicator studies (Cretì et al., 2010; Elnabris et al., 2013; Liang et al., 2024), the non-significant interaction for Hg points to a more uniform distribution. This could be due to the biomagnifying effects of methylmercury and its efficient transfer through trophic levels. The partial η^2^ values give quantitative information about the relative contributions of ecological and biological determinants. While interaction effects (η^2^_interaction_, 0.51–0.73) explained the largest proportion of variance for several metals, main effects were comparatively smaller (η^2^_species_, 0.03–0.18; η^2^_organ_, 0.02–0.13), supporting the combined influence of species-specific ecology and tissue-specific biochemical processes.

Comparable results have been reported in studies from the Arabian Sea and Bay of Bengal coasts, where Pb and Cd accumulation strongly correlated with species feeding habits and trophic levels (Lakshmanasenthil et al., 2013; Rainville et al., 2022; Shetye et al., 2014; Silambarasan, 2016). The results lend support to the assumption that the complex process of metal bioaccumulation involves exposure pathways, species ecology, and tissue-specific metabolism. Their utility in establishing localized contamination baselines for the protection of public health and fishery management is demonstrated by the observed patterns, which are largely consistent with global marine biomonitoring datasets.

## 4 Conclusion

The current study quantified the bioaccumulation of heavy metal(loids) in *Nemipterus japonicus*, *Oreochromis mossambicus*, and *Lates calcarifer*. The contamination level in *Nemipterus japonicus* was found to be in the order of Pb > Cr > Sr > Cd > Hg > V > As. Similarly, for *Oreochromis mossambicus* and *Lates calcarifer*, the contamination level was found to be in the following order: that is, Pb > Cr > Sr > Cd > Hg > V > As and Pb > Sr > Cr > Hg > Cd > V > As, respectively. The concentration was found to be in the range of 0.025–21.53 μg kg^−1^, considering all seven HMs.

The findings of this study are based on a limited sample size and do not account for temporal, spatial, or biological variations; therefore, the results should be interpreted as preliminary and indicative rather than population-representative.

With respect to non-carcinogenic risk, the THQ values remained <1 under the average seafood consumption scenario (150 g day^−1^ for adults; 75 g day^−1^ for children), suggesting low non-carcinogenic risk associated with the consumption of these species under the assumed exposure conditions.

For carcinogenic risk assessment, CR values for certain metals (notably As and Cr, and in some cases, Cd) exceeded the commonly referenced threshold of 1 × 10^−6^ under specific assumptions. The CR values estimated for chromium assume a conservative worst-case exposure scenario in which total chromium is considered as Cr(VI), potentially inflating cancer risk estimates and therefore requiring cautious interpretation. Under the average seafood consumption scenario, carcinogenic risk indices remained within acceptable regulatory thresholds for most of the metals analyzed; however, under higher consumption scenarios such as those observed in certain coastal or fishing-dependent communities, exposure levels may proportionally increase, particularly for vulnerable populations, including children and habitual seafood consumers.

These findings indicate that, while no immediate non-carcinogenic public health concern is suggested under average consumption assumptions, the theoretical carcinogenic risk associated with chromium warrants further investigation. Future studies with larger sample sizes, seasonal coverage, and chromium speciation analysis are recommended to better refine risk estimates. Apart from this, frequent monitoring of such environmental contaminants in food and other resources is highly demanded from a food safety and security perspective.

## Data Availability

The original contributions presented in the study are included in the article/Supplementary Material; further inquiries can be directed to the corresponding author.
